# An Atypical Presentation of Creutzfeldt-Jakob Disease With a Heidenhain Variant and Balint's Syndrome

**DOI:** 10.7759/cureus.8608

**Published:** 2020-06-13

**Authors:** Abhishek Gupta, Anurag Dhingra

**Affiliations:** 1 Geriatrics, Center for Addiction and Mental Health/University of Toronto, Toronto, CAN; 2 Family Medicine, Star Medical Center, Mississauga, CAN

**Keywords:** creutzfeldt jakob disease, neurocognitive dysfunction, dementia, atypical, cjd, heidenhain, balint's

## Abstract

Creutzfeldt-Jakob disease (CJD) is known for a rapidly progressive decline in cognitive functions due to an underlying infection from a prion. This case involves a unique and atypical variant of CJD that was difficult to diagnose for the investigating medical teams. As such, it highlights the particular challenge of using traditional diagnostic algorithms for atypical variants of CJD, in terms of time-appropriate diagnostics.

The patient presented with a sudden episode of vertigo which was treated as an isolated symptom. While succeeding investigations involving neurology and otolaryngology specialists were being carried out, the patient experienced progressively worsening and more frequent episodes of disequilibrium that required multiple visits to emergency care facilities. In such a facility, a repetitive battery of serum and cerebrospinal fluid testing confirmed the diagnosis of CJD. Subsequently, the patient was provided the necessary supportive care.

While this case was successfully diagnosed, it showed that common presentations can have significant underlying neurological implications, and such atypical variants should be accounted for in traditional diagnostic algorithms. This can avoid unnecessary delays in therapeutic rehabilitation.

## Introduction

Creutzfeldt-Jakob disease (CJD) is a spongiform neurodegenerative condition affecting various central nervous system (CNS) tissues [1]. It is the most prevalent prion disease in the world, with idiopathic, genetic and iatrogenic etiology [2]. Classic symptoms of CJD include rapidly progressive cognitive decline, ataxia and myoclonus. While visual symptoms are present in approximately 20% of the affected patients, the Heidenhain variant of the disease involves the same but exclusively at the onset [3].

Similarly, Balint's syndrome is a triad of simultanagnosia, ocular apraxia and optic apraxia, commonly associated with degeneration or injury to the parietal and occipital lobes. It is especially common in severe neurodegenerative disorders such as Alzheimer's disease [4].

This case report highlights the diagnostic challenge of incorporating advanced neurological diagnostic techniques into the diagnostic process while in the presence of initial symptoms that are classically not associated with neurological conditions. As such, it is also highlighted how such unusual presentations create diagnostic delays that can significantly hamper the appropriate treatment and rehabilitation.

## Case presentation

A 64-year-old male, with a history of controlled hypothyroidism and hypertension, initially arrived with an acute, limited episode of frontal headache characterized as severe frontal pressure with transient vertigo, lasting 15-20 seconds, when performing a calisthenic exercise.

Prior medical history included hypothyroidism controlled with levothyroxine 112 microgram (mccg) daily along with hypertension controlled with amlodipine 5 milligram (mg) daily, atorvastatin 40 mg daily and inhaled terbutaline sulfate for use as needed (PRN). The patient had quit smoking approximately three years ago. Family history was significant for ovarian cancer in his mother and congestive heart failure in his father. A review of travel and dietary history revealed extensive travel abroad to multiple destinations but no exposure to any exotic foods. He also admitted to significant stress due to his employment but denied any other psychiatric symptoms.

At the time, neurological examination, including the Romberg Test, were negative for any focal neurological deficits. However, apart from a hypertensive state (170/94), the other vital signs were within physiologic limits. As such, the patient was provided symptomatic treatment with beta-histine 16 mg orally (three times a day), to which, the client reported immediate relief.

Despite this temporary improvement, the episodic headaches recurred daily and progressed to include vertigo, vomiting and poor memory capacity. As the condition proved resistant to beta-histine, the method of symptomatic control was switched to chlorpromazine 10 mg daily. Within two weeks of the initial visit, the client developed severe visual symptoms, with disequilibrium, which prompted a referral to otolaryngology and neurology. These referrals were coincided with multiple emergency room visits for the same symptoms.

Specifically, the patient experienced episodic vertigo, fluctuating levels of blurred vision, bright lights, ataxia and temporary visual hallucinations. The patient reported seeing blue-yellow discolorations along with halos around otherwise normal appearing objects. This effect was especially pronounced when reopening eyes after prolonged closure. Extensive examination via the neurologist revealed a limited cognitive ability in visuospatial and executive function. On performing the Montreal Cognitive Assessment (MoCA), the patient scored 20 out of a maximum score of 30, particularly displaying difficulty with delayed recall, abstract thought and verbal fluency along with repetition. This was assessed to be a form of early onset dementia and follow-up cognitive testing was scheduled in three months.

During the neurological examination at this stage and on prior occasions, the patient displayed an otherwise normal neurological examination in reference to cranial nerve function, motor and sensory function, coordination and gait.

Serum testing for a complete blood cell count showed a mildly low red blood cell count at 4.59 g/dL (Normal range (n): 4.7-6 g/dL) while mildly elevated levels of platelet sat 401 x 10^9^/L (n: 150-400 x 10^9^/L) and monocytes at 0.9 x 10^9^/L (n: 0.0-0.8 x 10^9^/L). A serum electrolyte panel showed mildly low sodium level at 135 mmol/L (n: 136-144 mmol/L). Follow-up serum testing identified other abnormalities including an elevated thyroid peroxidase antibody titre of 59 (n: 0-34), a low serum uric acid level at 248 umol/L (n: 286-518 umol/L) along with elevated levels of total bilirubin at 28 umol/L (n: 3-17 umol/L) and direct bilirubin at 6 umol/l (n: 0-5 umol/L). He also had consistent hyperglycemia on two occasions six weeks apart at 7.4 mmol/L and 7.6 mmol/L (n: 4-6 mmol/L), respectively. Otherwise, the patient had a normal urinalysis, coagulation profile and serum protein electrophoresis.

Follow-up assessments with an otolaryngologist, an audiologist, and an ophthalmologist were negative for any significant abnormalities. During an ensuing emergency neurological assessment, the patient had developed severe vertigo, oscillopsia, dyschromatopsia, visual agnosia, simultanagnosia and right homonymous hemianopsia, requiring inpatient care. This cluster of oculomotor symptoms conformed to a diagnosis of Balint's syndrome.

A computed tomography (CT) scan of the head and neck (without contrast) was normal but an electroencephalogram (EEG) indicated several diffuse abnormalities. The EEG (conducted while awake and occasionally in light sleep) showed a poorly organized background, often reaching the alpha range (8-9 Hz, medium amplitude and fairly symmetrical). This was interrupted by slower delta and theta range waveforms, often sharply contoured. In the frontal head region, especially during drowsiness, high amplitude delta activity appeared in semirhythmic/polymorphic fashion (medium to medium-high amplitude in moderate amounts). Photic stimulation resulted in no EEG changes and hyperventilation trial was not conducted. These patterns conformed to the Heidenhain variant of CJD.

Further testing on the cerebrospinal fluid (CSF) confirmed the diagnosis via a positive real-time quaking-induced conversion (RT-QuIC) assay. Also, a positive enzyme-linked immunofluorescence (ELISA) assay for Tau protein and 14-3-3 protein contributed to this diagnosis.

The patient was discharged after a 10-day inpatient stay after his symptoms were stabilized, with an outpatient palliative care plan in place. His medications included levothyroxine 0.112 mg orally once per day, inhaled budesonide 200 microgram (mcg) per dose once daily, aluminum and magnesium hydroxide 10-20 mL orally every 4 hours (hrs) and lorazepam 1 mg sublingual as needed for agitation and insomnia.

The patient's cognition continued to deteriorate despite extensive outpatient palliative care. Home-based assessments indicated the patient required considerable assistance with ambulation and other activities of daily living (ADLs), which also contributed significant mental stress. A trial of percutaneous endoscopic gastrostomy (PEG) was complicated with severe diarrhea and mild hematochezia, requiring an emergency transfer to an inpatient palliative care unit. Clostridium Difficile (C. Diff.) cultures on stool samples was negative. The patient ultimately succumbed due to extensive gastrointestinal hemorrhage.

## Discussion

This case highlights a significant challenge towards the diagnosis of CJD, especially considering the heterogeneity and atypical nature of its manifestations presented here.

Given the patient’s initial vestibular symptoms along with an otherwise normal neurological exam, a lack of any specific focal neurologic signs, and an initially intact memory, vestibulocochlear pathology instead of neurocognitive function was the focus of initial investigations. As such, the patient was referred for otolaryngology, audiology and ophthalmology assessments. Only with the onset of visual hallucinations and the persistent, progressive nature of the disequilibrium, a neurological pathology was suspected.

Even with the initial neurological assessment discovering an early-onset neurocognitive dysfunction, the relatively mild state did not necessitate an urgent serological and cerebrospinal fluid (CSF) analysis. However, following the rapid decline in the patient’s cognitive function and cerebellar symptoms which led to an emergency CSF analysis and the subsequent abnormal EEG pattern, the underlying CJD was coincidentally diagnosed.

The diagnostic criteria for neurocognitive disorders require a cognitive decline in at least one cognitive domain; complex attention, executive function, language, learning and memory, perceptual-motor, or social cognition (Table 1) [5]. Considering that the patient exhibited some limited ability in visuospatial and executive function during the initial neurological assessments, without any visible effect on activities of daily living (ADLs), the patient suffered from mild cognitive impairment (MCI) [6]. Subsequently, as Shaw et al. have shown, mild cognitive impairment or dementia with an onset at an early age (<65) merits a lumbar puncture (LP) and CSF testing [7].

**Table 1:** Diagnostic criteria for neurocognitive disorders Table adapted from [5], Page 400

Major Neurocognitive Disorder	Minor Neurocognitive Disorder
Significant cognitive decline in at least one cognitive domain as seen in both of the following:	Modest cognitive decline in at least one cognitive domain as seen in both of the following:
Concerns expressed by the patient or reliable informant or as seen by the clinician	Concerns expressed by the patient or reliable informant or as seen by the clinician
Objective neurocognitive testing/assessments	Objective neurocognitive testing/assessments
Interference with instrumental activities of daily living	Does not interfere with instrumental activities of daily living, but they require additional time and effort

The following criteria apply to both major and minor neurocognitive disorders:
Cannot occur exclusively during bouts of delirium
Cannot be explained by another mental disorder
Specify one or more causal subtypes:
Alzheimer Disease
Frontotemporal lobar degeneration
Human Immunodeficiency virus infection
Huntington Disease
Lewy body Dementia
Parkinson Disease
Prion disease
Substance/medication use
Traumatic brain injury
Vascular disease
Other Medical Condition
Multiple etiologies

Consequently, the aim of the early neurological assessments was to monitor the patient for a pattern of continued or worsening cognitive impairment while vestibulocochlear pathology was excluded in order to recommend CSF analysis. However, despite the client meeting the diagnostic criteria for possible CJD dementia (Table 2), the sudden and rapid cognitive decline had not been anticipated in the framework of the timeline for advanced neurological testing [8]. As the CJD course lasts for eight months on average and 90% of the patients die within one year on onset of symptoms, the delayed diagnosis had a limited effect on the overall prognosis of the patient’s condition [1].

**Table 2:** MRI-CJD Consortium criteria for sporadic Creutzfeldt–Jakob disease Table adapted from [8], Page 2664 PSWC: Periodic Sharp Wave Complexes; EEG: Electroencephalogram; CSF: Cerebrospinal Fluid; DWI: Diffusion-Weight Imaging; FLAIR: Fluid Attenuated Inversion Recovery; CJD: Creutzfeldt-Jakob Disease; MRI: Magnetic Resonance Imaging.

Classical Signs and Tests:

I. Classical Signs
Dementia
Cerebellar or Visual
Pyramidal or Extrapyramidal
Akinetic mutism

II. Tests
PSWCs in EEG
14-3-3 detection in CSF (in patients with disease duration of less than two years)
High signal abnormalities in caudate nucleus and putamen or at least two cortical regions (temporal-parietal-occipital) either in DWI or FLAIR

MRI-CJD Consortium Criteria:
Probable CJD – Two of (I) and at least one of (II)
Possible CJD – Two out (I) and duration less than two years

## Conclusions

The Heidenhain variant of CJD is a vestibulocochlear symptomatic presentation hiding severe and rapidly developing underlying neurological sequelae. As such, it is important to establish guidelines for atypical presentations of CJD’s cognitive impairment that incorporate appropriately timed CSF analysis or advanced cognitive assessments. While such analysis has a limited effect on the overall prognosis, given the terminal nature of CJD, it is critical towards the overall accurate diagnosis and rehabilitation.

Atypical presentations, by definition, are challenging because they defy the traditional algorithmic approach to medical diagnosis. This challenge is further compounded when there is no evidence of risks and imaging technology is of limited use. This case highlights the significant delays that can be brought on by such atypical scenarios and the necessary research on neurological diagnostic protocols to anticipate them.

## References

[1] Proulx AA, Strong MJ, Nicolle DA (2008). Creutzfeldt-Jakob disease presenting with visual manifestations. Can J Ophthalmol.

[2] Verma R, Junewar V, Sahu R (2013). Creutzfeldt-Jakob disease presenting with visual symptoms: a case of the 'Heidenhain variant'. Case Rep.

[3] Wong A, Matheos K, Danesh-Meyer H (2015). Visual symptoms in the presentation of Creutzfeldt-Jakob disease. J Clin Neurosci.

[4] Ghoneim A, Pollard C, Greene J, Jampana R (2018). Balint syndrome (chronic visual-spatial disorder) presenting without known cause. Radiol Case Rep.

[5] Falk N, Cole A, Meredith TJ (2018). Evaluation of suspected dementia. Am Fam Physician.

[6] Gillis C, Mirzaei F, Potashman M, Ikram MA, Maserejian N (2019). The incidence of mild cognitive impairment: a systematic review and data synthesis. Alzheimers Dement (Amst).

[7] Shaw LM, Arias J, Blennow K (2018). Appropriate use criteria for lumbar puncture and cerebrospinal fluid testing in the diagnosis of Alzheimer's disease. Alzheimers Dement.

[8] Zerr I, Kallenberg K, Summers DM (2009). Updated clinical diagnostic criteria for sporadic Creutzfeldt-Jakob disease. Brain.

